# Editorial: Eye in systemic diseases

**DOI:** 10.3389/fmed.2023.1171238

**Published:** 2023-03-24

**Authors:** Anna M. Roszkowska, Paolo Fogagnolo, Piergiorgio Neri

**Affiliations:** ^1^Ophthalmology Clinic, Department of Biomedical Sciences, University of Messina, Messina, Italy; ^2^Ophthalmology Clinic, Faculty of Medicine and Health Sciences, Andrzej Frycz Modrzewski Kraków University, Kraków, Poland; ^3^Ophthalmology Clinic, University of Milan, Milan, Italy; ^4^The Eye Institute, Cleveland Clinic Abu Dhabi, Abu Dhabi, United Arab Emirates; ^5^Cleveland Clinic Lerner College of Medicine, Case Western Reserve University, Cleveland, OH, United States; ^6^College of Medicine and Health Sciences, Khalifa University, Abu Dhabi, United Arab Emirates

**Keywords:** systemic diseases, ocular diseases, diabetic retinopathy, dry eye, OCT, IVCM

The systemic diseases might involve the eyes in many ways, either with typical signs and symptoms or, often, with atypical presentation.

A rapid diagnosis may promote prompt treatment and a better clinical outcome in different cases.

Ocular involvement is present in metabolic, vascular, and rheumatologic diseases with retinopathy, inflammation, ocular surface, and corneal involvement. It may also be observed in different genetic syndromes. On the other hand, several systemic therapies may induce ocular changes, potentially affecting visual acuity.

This Research Topic aims to provide as comprehensive as possible information on ocular changes in systemic diseases, the signs, and symptoms that should be considered when systemic diseases are suspected. Additionally, it aims to evidence the ocular involvement in systemic disease and to highlight the necessity of the multidisciplinary approach for diagnosis and treatment.

Diabetes is undoubtedly the most common disease, with increasing prevalence worldwide, that involves different organs and tissues with diabetic angiopathy and neuropathy. It affects the eye with many degrees of severity diabetic retinopathy (DR) and cornea with tiny fibers neuropathy that affects the corneal sub-basal nerve plexus and may lead to severe visual impairment. In addition, it was reported that DR could be associated with cardiovascular involvement and stroke (1, 2).

Barrot et al. evaluated the role of diabetic retinopathy as a predictor of cardiovascular morbidity and mortality in subjects with type 2 diabetes in the Catalonia (Spain) population.

The authors investigated the predictive value of DR with its severity with the incidence of major cardiovascular events such as coronary heart disease, stroke, and all-cause mortality in subjects with T2DM in a Mediterranean region. They concluded that DR is related to coronary heart disease, macrovascular events, and all-cause mortality among persons with T2DM. The authors highlighted the importance of prompt screening and proper treatment of diabetic patients with DR to avoid cardiovascular complications leading to death.

Continuing with diabetes, another paper focused on the association between serum magnesium levels and diabetic macula edema (DME) in patients with DR (Xiang et al.). The systemic conditions that result from reduced serum magnesium might worsen the DR and promote DME with severe visual impairment (3).

The authors demonstrated that a higher serum magnesium level was associated with a lower risk of DME in patients with DR. These data open a new Research Topic to assess whether appropriate magnesium supplementation in diabetic patients reduces the risk of DME.

The relation between DR and Parkinson's disease (PD) as diabetes with DR and PD share some pathophysiological mechanisms related to stopped dopamine activity as both brain and retina are characterized by expression of D1like and D2-like dopamine receptors. So, the recognition of the DR as a marker of PD was hypothesized (4).

Mauricio et al. evaluated the primary health care large population in Catalonia (Spain) with type 2 diabetes and diabetic retinopathy for the risk of occurrence of PD. The authors concluded that DR was not associated with an increased risk of PD after adjusting for different risk factors such as age, male sex, and diabetes duration.

Dry eye is a multifactorial ocular surface disease affecting up to 50% of the population, significantly impacting the quality of life. Several risk factors for developing the disease and sleep apnea may dramatically impact ocular surface conditions (5). Pu et al. demonstrated a higher prevalence of DED in patients with obstructive sleep apnea syndrome (OSA), with a significant correlation between DED parameters worsening and OSA severity. This evident interaction should be addressed in patients with OSA, and proper ocular surface therapy should be considered.

Severe cornea and ocular surface disease are represented by neurotrophic keratitis (NK). Several systemic diseases, such as diabetes, rheumatoid arthritis, and atopia, might produce NK with severe visual impairment. Therefore, the patients should be monitored to promptly diagnose the early stage of corneal involvement to start the appropriate therapy to avoid, if possible, the progression that may lead to visual loss (6–10). In the paper related to NK in systemic diseases, Meduri et al. evidenced that the leading cause of NK was post-neuroma surgery (36%), followed by diabetes (18%). The remaining causes were rheumatoid arthritis (9%), post-traumatic (9%), post-surgery (9%), atopic (9%), and Graves' disease (9%). Additionally, the results of therapy with rh-NGF (Cenegermin) were presented, and the authors concluded that current knowledge of the pathogenesis of NK and the introduction of topical recombinant human Nerve Growth factor (rh-NGF) has significantly changed the natural history of the disease.

The effects of smoking on the microvascular system result in ocular complications (11).

The exciting paper of Xu et al. on the impact of chronic smoking on the microvascular system demonstrated the damage to the retinal vascular system. Furthermore, the authors highlighted the role of prevention and lifestyle improvement in preventing systemic diseases.

The research and validation of the new accurate and reliable tools to be used in the screening and assessment of systemic diseases are of extraordinary actuality, primarily due to the remarkable technological progress and availability of instruments increasingly sophisticated.

Retinal changes in different neurodegenerative diseases suggest its parallel involvement with significant differences. Therefore, the retina was proposed as the window to the neurodegenerative changes in the central nervous system (12).

For this purpose, Deng et al. performed a systematic review and meta-analysis to evaluate retinal and microvascular parameters in patients with PD as compared to healthy controls. The authors considered RNFL, macular, GCL, vessel density, and optic disk area evaluated using OCT. The study evidenced that studied parameters were significantly lower in PD patients confirming that OCT and OCTA might play a role in detecting early morphological retinal changes in patients with PD and consequently support clinicians in diagnostic processes.

Additionally, OCT can classify PD patients accordingly to measure retinal changes. The authors speculated that in the next future, the OCT and OCTA might be used to assess the progression of PD based on variations of retinal parameters.

The last decades of exceptional technological progress have offered clinicians and researchers new diagnostic tools for visual disease assessment and follow-up.

And while OCT continuously improved and became essential for retinal examination, in corneal semiotics, AS-OCT and IVCM are now fundamental. Moreover, the use of IVCM to diagnose systemic diseases by corneal SBNP examination has become of growing interest, and its use in the assessment of diabetic peripheral polyneuropathy was widely documented (13–16).

Gu et al. performed a review of corneal confocal microscopy in the assessment of non-neurological autoimmune diseases. The authors concluded that IVCM parameters were altered in patients with NNAI affections compared to healthy subjects and highlighted the role of IVCM in early diagnosis and follow-up of affected patients.

The research to develop a more accurate analysis of IVCM data is the topic of the paper of Abicca et al., who presented the new algorithm for the evaluation of corneal nerves beadings in diabetic patients using IVCM. This new evaluation method adds a further possibility to investigate nerves changes in the early stage of peripheral neuropathy.

Lombardo et al. reports retinal imaging with new devices and a new approach to AMD.

Age-related macular degeneration is a visual threatening multifactorial disease with several systemic disorders such as hypertension, overweight, and low dietary intake of carotenoids act as decisive risk factors (17). In their report, the authors present a new system providing topical delivery of lutein into the retina using iontophoresis and show promising results of the pilot study on patients with AMD (18, 19). Furthermore, the authors discuss the advantage of using adaptive optics technology to improve the performance of optical systems by reducing the effects of optical distortions. Consequently, the improved resolution provides a more sensitive tool to study, detect, and track retinal diseases. Additionally, they present Resonance Raman spectroscopy (RRS) as one of the most promising technologies for measuring macular carotenoid levels from the human retina (20, 21).

Ocular adverse effects of systemic therapies were the topic of some papers.

The use of dupilomab, a targeted biological drug for atopic dermatitis (AD), is widely performed in adults and children. But the adverse effects could be expected, and they manifest with ocular surface diseases (22). Jia et al. reviewed ocular adverse effects in patients treated with dupilumab for atopic dermatitis. The AE associated with the therapy manifested in up to 50% of patients with non-infectious conjunctivitis, followed by ocular pruritus, blepharitis, xerophthalmia, and keratitis. The cause is attributable to the inhibition of goblet cells through blocking IL-4 and IL-13 with dupilumab, which results in reduced mucin secretion. Another mechanism of conjunctivitis may be associated with serum IgE, thymus, and activation-regulated chemokine in dupilumab-treated patients (23). The authors reported good treatment results with fluorometholone or tacrolimus in affected patients providing dermatologists and ophthalmologists with diagnostic and therapeutic recommendations.

The widespread use of local and systemic corticosteroids might be controversial, and accurate estimation of risk and benefits should be consistently done (24). This problem emerges from the case report on the role of corticosteroids in treating acute ocular toxoplasmosis in an immunocompetent patient (25). Lin et al. showed that the early use of systemic corticosteroids in patients with acquired ocular toxoplasmosis might induce severe retinal visual-threatening complications. Therefore, they recommend accurate and continuous visual monitoring during therapy.

In conclusion, this special issue includes papers that provide information on ocular involvement in systemic disease that should be promptly diagnosed and treated. The multidisciplinary approach to diagnose, treat, and monitor the patients is recommended. Additionally, the issue highlights new possibilities to diagnose and follow up different systemic diseases by ophthalmic evaluation using the new examination tools. We hope that the content of this special issue will raise the level of understanding how the systemic diseases might impact on ocular health.

## Author contributions

AR and PF drafted and wrote the paper. PN revised and wrote the paper. All authors contributed to the article and approved the final version.
